# Design and Implementation of a GPS Guidance System for Agricultural Tractors Using Augmented Reality Technology

**DOI:** 10.3390/s101110435

**Published:** 2010-11-18

**Authors:** Javier Santana-Fernández, Jaime Gómez-Gil, Laura del-Pozo-San-Cirilo

**Affiliations:** 1 R&D Department, Agroguia, Alaejos, 47510 Valladolid, Spain; E-Mail: laura@agroguia.es; 2 Department of Signal Theory, Communications and Telematics Engineering, University of Valladolid, 47011 Valladolid, Spain; E-Mail: jgomez@tel.uva.es

**Keywords:** augmented reality, virtual reality, guidance, GPS, precision farming

## Abstract

Current commercial tractor guidance systems present to the driver information to perform agricultural tasks in the best way. This information generally includes a treated zones map referenced to the tractor’s position. Unlike actual guidance systems where the tractor driver must mentally associate treated zone maps and the plot layout, this paper presents a guidance system that using Augmented Reality (AR) technology, allows the tractor driver to see the real plot though eye monitor glasses with the treated zones in a different color. The paper includes a description of the system hardware and software, a real test done with image captures seen by the tractor driver, and a discussion predicting that the historical evolution of guidance systems could involve the use of AR technology in the agricultural guidance and monitoring systems.

## 1. Introduction

Virtual Reality (VR) is a technology that presents a synthetically generated environment to the user though visual, auditory and other stimuli [1]. It uses hardware such as head mounted displays and employs 3D processing technologies. The areas where VR had been used are many, including among others e-commerce [2], manufacturing [3], the military [4], medicine [5] or education [6].

By contrast, Augmented Reality (AR) is a VR-like technology that presents to the user synthetically generated information superimposed onto the real world [7]. That is, AR is an expansion of the real world achieved by projecting virtual elements onto it [8]. AR simply enhances the real environment, whereas VR replaces it. AR can be utilized in areas such as urban and landscape planning [9], manufacturing [10], medicine [11], defense [12], tourism [13] and education [14], among others.

In the agricultural field scientific literature some authors have presented works employing VR over Geographics Information Systems (GIS), see for example Lin *et al.* [15] and Rovira-Mas *et al.* [16]. These papers present systems to acquire information from the environment with stereovision devices, store the information in a GIS system and then reconstruct the environment with 3D visualization. The scientific literature that employs AR in agriculture is more reduced, see for example Min *et al.* [17] and Vidal *et al.* [18].

We have developed and tested an agricultural guidance system that uses AR technology. This paper presents this system and the visual results obtained. Moreover, in the Discussion section, this paper presents the evolution of guidance techniques in agriculture, which predicts the use of AR technology in future agricultural systems.

## 2. Description of the Guidance System

The guidance system is composed of a software application that runs over a hardware system. The application acquires information from some hardware sensors, processes this information and presents a video of it on *eye monitor glasses*. The hardware and software of this system are described in this section.

### 2.1. System Hardware

**Figure 1.**
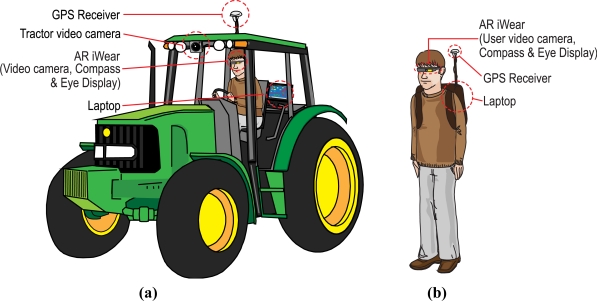
Hardware of the system. (a) “Tractor equipment”, the hardware of the system when the user is inside the tractor cab. (b) “Exterior equipment”, the hardware of the system when the tractor driver is outside the tractor cab.

There is a hardware implementation that is employed when the user is driving the tractor, which we call “tractor equipment”. There is another hardware implementation employed when the user is walking around the land. We call it “exterior equipment”. Figure 1 shows the components and disposition of both implementations.

A GPS receiver was used for positioning the user on the land and, therefore, to locate the video camera that obtains the user’s vision field. A *Novatel Smart Antenna V1* that provides 5 Hz update positioning frequency was used in the experiments of this paper.

A *Vuzix iWear VR 920* eye display was employed to present to the user the information generated by the developed guidance system. This device integrates a *3*-*Degree of Freedom* (DOF) digital compass.

A *Genius VideoCAM Slim USB2* camera attached to the tractor cab was employed to obtain the user’s vision field when he was driving the tractor. A *Vuzix CamAR* video camera attached to the eye display was employed to obtain the user’s vision field when he was outside the tractor. Finally, a *DELL Inspiron 6400* laptop processes the data in real time.

### 2.2. System Software

**Figure 2.**
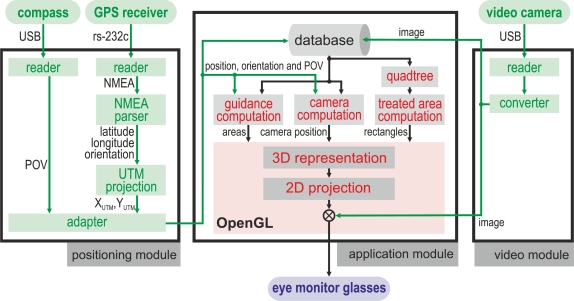
This figure shows the diagram of the application modules. There are three main modules. The central one processes the information provided by the positioning and video camera modules to generate the 2D projection over the real time video acquired.

The software of the system was developed in *Python* programming language and was run on a laptop equipped with the *Microsoft Windows XP* operating system. The application was subdivided into three main modules, according to Figure 2.

The *positioning module* deals with the user and tractor positioning. It reads and parses the NMEA sentences from a GPS receiver to obtain latitude, longitude, orientation and time data. After this, it converts the geographical coordinates received from the GPS to the *Universal Tranverse Mercator* (UTM) coordinate system. This module also obtains the *Point of View* (POV) of the video camera that records the video, which is composed by its position coordinates and by its orientation vector.

The *video module* gets a video sequence from the *Vuzix CamAR* video camera attached to the eye display or from the *Genius VideoCAM Slim USB2* video camera placed over the tractor cab.

The *application module* receives data from *positioning* and *video modules*, gets data from the database, processes the data and outputs a video sequence through the eye display. More concretely, this module performs the following tasks: (i) the latest position and orientation data are stored in the database, (ii) the camera orientation and location are achieved, (iii) the 2D map and camera frustums are computed, (iv) the threaded zone rectangles are obtained from the database using quadtree, (v) the guidance lines are computed and (vi) all the 3D data and camera images are sent to *OpenGL* to be rendered on the eye display.

### 2.3. Three Dimensional Information Management

The developed system shows information superimposed on captured real world video images. For that reason, it needs to process the data and render 3D information to match the real video. *OpenGL* is a rendering *Application Programing Interface* (API) that allows rendering of 3D information such as rectangles, lines and other primitives and projects it onto a 2D screen.

The treated zone is displayed using small rectangles that approach the tractor path. Each rectangle has position information formed by the x and y position in UTM projection, the z = 0 plane, the working width and the heading vector. The vertices of the rectangle are computed with this information and passed to the *OpenGL* API to be rendered as rectangle primitives. In the same way, the guidance lines are specified to *OpenGL* using line primitives. Spatial information, color, transparency and other rendering parameters are indicated to *OpenGL* to render those primitives.

*OpenGL* has an API to locate a camera in the 3D world that represents the observer’s POV. The camera needs position, direction and an up vector. Camera position is obtained from GPS position using the UTM projection. Direction and up vector are obtained in two ways: (i) in the tractor equipment, these vectors are obtained from GPS heading and the camera placement angles in the tractor, and (ii) in the exterior equipment these angles are provided by the 3 DOF compass integrated into the *Vuzix iWear VR 920*. Readers can obtain more information about three dimensional information management in the *The Official Guide to Learning OpenGL* [19].

### 2.4. Two Dimensional Projection

**Figure 3.**
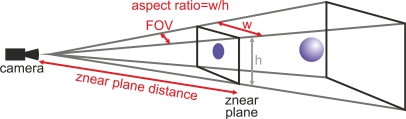
Camera view fustum showing in red the camera definition parameters: *horizontal FOV angle*, *znear plane distance* and *aspect ratio*.

The computer screen is a 2D visualization device. In order to display 3D information on it, the application needs to project this data over a 2D plane. *OpenGL* provides an API to perform this projection. The API uses three real camera parameters: *horizontal Field Of View* (FOV) *angle*, *aspect ratio* and *znear plane distance* (see Figure 3). Board *et al.* explain in detail this projection process using *OpenGL* [19].

### 2.5. Camera Calibration

The camera definition parameters of the user camera *Vuzix CamAR* were specified by the camera manufacturer in its user manual. They are *horizontal FOV* angle = 32°, *znear plane distance* = 0.1 m and *aspect ratio* = 1.33.

The camera definition parameters of the *Genius VideoCAM Slim USB2* tractor camera employed were not provided by the manufacturer. For this reason it was necessary to compute them experimentally. It was done following the next three steps: initially a real scene was built with basic objects whose size and placement were known. Next, the same scene was modeled in 3D with the camera location and orientation known and the object sizes and placements also known. Finally, the parameters were manually modified in the application until the real and 3D modeled scenes matched on the screen.

**Figure 4.**
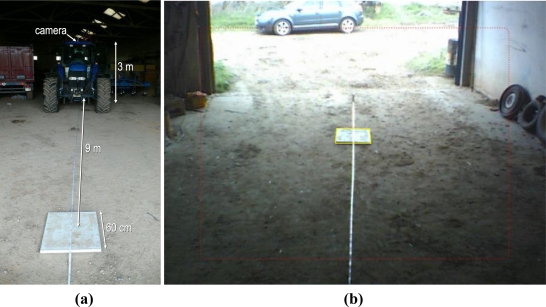
(**a**) Photo of the tractor and the object placed to calibrate the tractor video camera. (**b**) The representation, in yellow, of the 3D modeled square over the real image during the process of getting the camera parameters.

Figure 4 shows the placement of a square object in a well-known location in order to calibrate the tractor video camera, which was placed over the tractor cab, and the representation of the 3D modeled square over the real image in the process of getting the camera parameters. The camera parameters obtained for the *Genius VideoCAM Slim USB2* were *horizontal FOV* angle = 42°, *znear plane distance* = 0.1 m and *aspect ratio* = 1.33.

### 2.6. Guidance

In agricultural tasks, tractors usually need to follow a trajectory equidistant to a previous pass. Using GPS guidance systems, this can be accomplished through an autonomous guidance system or through an assisted guidance system.

Autonomous guidance systems employ control laws to steer the tractor taking into account two inputs; (i) the distance of the tractor to the desired trajectory, and (ii) the orientation difference between the tractor and the nearest point to the tractor in the trajectory. A typical control law used in high precision guidance systems is:

(1) $$\delta =\arctan\left((-k_{1}\cdot x-k_{2}\cdot \text{tan}\;\theta )L\cdot \cos^{3}\theta \right),$$

where *δ* is the steering angle, *x* is the tractor distance to the desired trajectory, *θ* is the orientation difference between the tractor and the trajectory in the nearest point to the tractor, *L* is tractor distance between axes and k_1_ and k_2_ are two constant control gains [20–22].

Assisted guidance systems work in a similar way, but they do not steer the wheel. Instead, they show the tractor driver information about the amount and direction the steering wheel should be moved to follow a desired trajectory. Usually this information is provided by means of a light bar or by an on-screen bar.

In the assisted guidance system developed here, this information was put in the screen by means of a quantity of arrows. This arrow quantity was computed using a fuzzy logic control law, which was experimentally tuned.

### 2.7. Spatial Information Management

Tractor guidance systems need to store the complete trajectory followed by the tractor in the agricultural task. This trajectory consists of mainly the tractor positions received by the positioning system, in our case just the GPS receiver. When a tractor guidance system is working, some new positions must to be stored every second. In our system, this number was five, because we employed a GPS receiver with a 5 Hz update frequency.

Tractor guidance systems need to continuously compute the steering turn and the treated zones map. To do this, it is necessary to process the tractor fragment trajectories near the spatial position of the tractor in each time instant, but taking into account the usual trajectory followed by a tractor working in a plot, it is easy to perceive that some trajectory points near in space are far in time. Due to this, the spatial points of the trajectories must be stored in the guidance system memory following spatial data structures. These structures allow access to all trajectory points near in space to the spatial tractor position.

The spatial data structure employed in the developed guidance system was a quadtree [23]. In the data structure were stored objects with a GPS position and information about the spatial area treated by the tractor in the last time interval. Each object was stored in a node with the structure: class Quadtree{Quadtree* _child[4];ObjectList _objectList;BBox _bbox;int _depth;};where:

_child is a four pointers vector to four child nodes. Nodes without children have the zero value.

_objects is a list of pointers to objects stored in the structure. When this list reaches 16 pointers, four child nodes are created and objects of father node are distributed between child nodes. The maximum depth of the quadtree is 11 levels, because more levels increase the insert and extraction times. These numbers, 16 and 11, were obtained with simulations using real paths followed by a tractor working in plots.

_bbox contains geometric information about the objects of the node in order to speed up processing time. It contains the four vertices that include all objects of the node.

_depth is a number that represents the node depth in the quadtree structure. Fader node has 0 value, children from father has 1 value. Children from children of father have the 2 value and so on.

**Figure 5.**
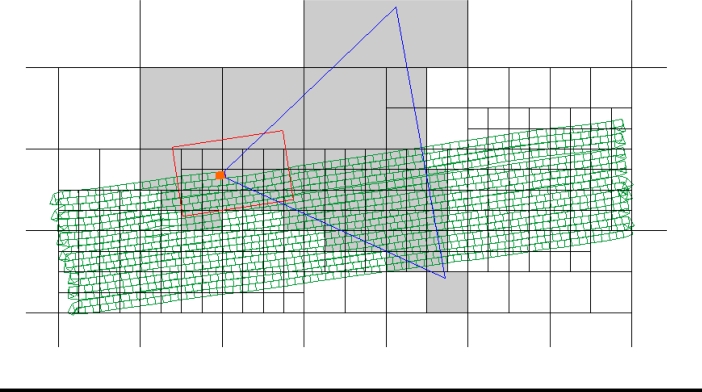
Screen capture of a quadtree visor developed to preview the quadtree splitting.

Figure 5 shows a screen capture of a quadtree visor developed to preview the quadtree splitting. In this figure, the black lines represent the node divisions. The green polygons represent the surface units treated by the tractor along its trajectory. The orange polygon represents the last surface unit covered by the tractor. The red rectangle represents the 2D map frustum and the blue polygon represents the camera frustum. In the instant time corresponding to Figure 5, only the nodes placed below one or both frustums, which are in grey color, must be processed to present the user with the maps of treated areas.

### 2.8. Google Earth Integration

**Figure 6.**
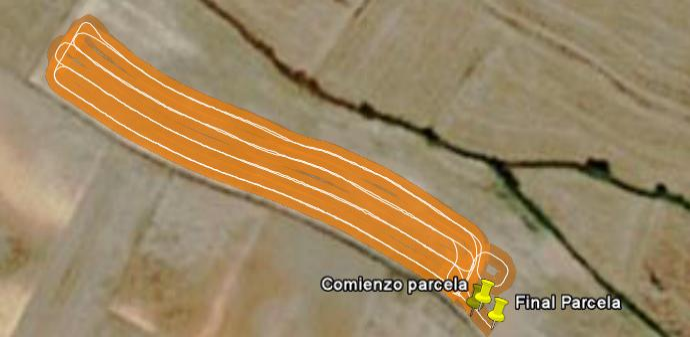
Visualization with *Google Earth* of the trajectories and treated zones in the real test.

The guidance system was developed using software components from the Spanish commercial tractor guidance system *Agroguia*^®^. Due to this, *Agroguia*^®^ and the developed system can export the geographic information of the work in a *.kml file that can be opened by the free geographic information program *Google Earth*. In this way, as Figure 6 shows, the trajectories, the treated zones and some other information can see on orthophotos using *Google Earth*.

## 3. Results

The developed system allows the tractor driver to receive the guidance information in a very natural way. Thanks to the augmented reality, the tractor driver can see over the land scenery some information about the treated and non-treated areas. The developed system also provides (i) the speed and total area values, (ii) a two-dimensional map of the treated areas and (iii) some arrows that provide the driver with information about the magnitude and sense of turning the steering wheel in order to follow the correct trajectory.

**Figure 7.**
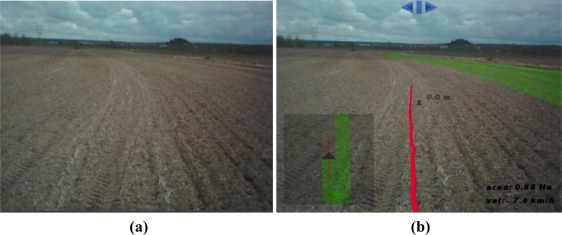
A possible scene that the tractor driver sees: (**a**) without the developed system or (**b**) with the developed augmented reality system.

**Figure 8.**
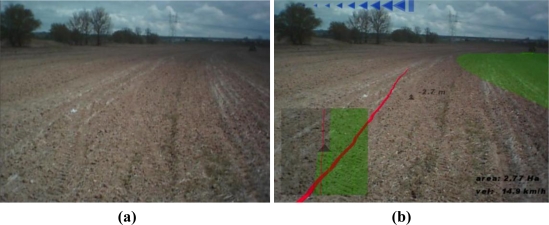
Another possible scene that the tractor driver sees: (**a**) without the use of the developed system or (**b**) with the use of the developed augmented reality system.

Figure 7 shows a situation with the tractor correctly following the desired trajectory. Figure 8 shows another situation where the tractor driver needs to turn the steering wheel to the left, because the tractor implement is overlapping the previous pass.

When the user was driving the tractor, the user camera captured the front wheels and the frontal parts of the tractor, the upper part or the steering turn and some other parts of the tractor cab. The map superimposed over these images produced a strange sensation. For this reason, processed images of the land (Figure 7 and Figure 8) were generated from video captured with the tractor video camera placed over the tractor cab, for presentation in this document and to the tractor driver.

**Figure 9.**
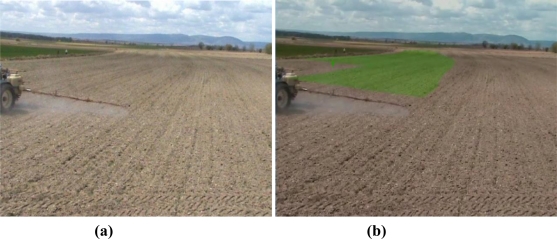
A possible scene that an observer sees: (**a**) without the developed system or (**b**) with the developed augmented reality system.

**Figure 10.**
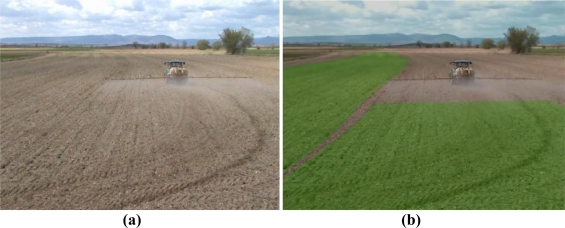
Another possible scene that an observer sees: (**a**) without the use of the developed system or (**b**) with the use of the developed augmented reality system.

The developed system also allows any observer to see the treated areas in the plot. Figure 9 and Figure 10 show the experience obtained by an external observer standing in a high place, wearing the *Vuzix iWear VR 920* eye display with the *Vuzix CamAR* video camera attached to it.

## 4. Discussion

**Figure 11.**
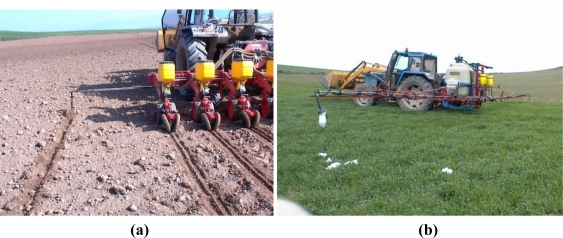
(**a**) A seeder equipped with a mechanical marker, which produces a trace to be followed by the tractor in subsequent rounds (**b**) A tractor with a spraying machine that produces foam flakes to demarcate the treated zone.

Some agricultural tasks, such as plowing or harvesting, produce changes in the color or in the texture of the treated areas, but no visual effects are produced by other tasks, such as fertilizing or spraying. Moreover, agricultural tasks are continually increasing their working width and this makes tractor driving difficult. Due to this, different strategies are used by farmers in order to guide the tractor along the plot, when a visual guidance cannot be easily performed. One of them is the use of mechanical markers, which produce a trace to be followed by the tractor in subsequent rounds [see Figure 11(a)]. Another is the use of foam markers, used mainly in spraying or fertilizing applications. This type of marker produces foam flakes to demarcate the treated zone [see Figure 11(b)].

**Figure 12.**
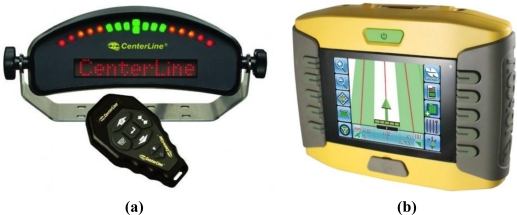
(**a**) Control panel of the *Centerline Guidance Lightbar* marketed by *Teejet* [24]. (**b**) Control panel of the *150 Tractor Guidance System* manufactured by *Topcon* [25].

The development of GPS technology and the reduction in price of the necessary receivers has made possible their use in different sectors. One of these is agriculture, where nowadays GPS technology is widely used to assist in the guidance of tractors and other agricultural vehicles or even to autonomously guide them. The information offered to the tractor driver by commercial tractor guidance systems can be categorized into two types:

- Guidance systems that only provide information about the magnitude and sense of turning the steering wheel in order to follow the correct trajectory. They are commonly named *lightbar* guidance systems because most of these types of devices consist of LED diodes placed in a horizontal line. The first guidance tractor systems were of this type, but nowadays they have largely fallen into disuse. Figure 12(a) shows a device of this type.
- Guidance systems with a wide screen that can display much more information, including a map of the treated areas referenced to the tractor position. The screen usually offers touch control possibilities, and some of these guidance systems are integrated in the tractors by the tractor manufacturers. Figure 12(b) shows a device of this type.

Mechanical markers, foam markers, or the *lightbar* GPS guidance systems, are all valid options to guide the tractor when no visual marks are produced in the agricultural task. But the tractor driver must make the effort to mentally create, actualize, and retain, a map of treated areas. This mental effort could be considerable in irregularly shaped lands.

Guidance systems with a screen that provides a map of treated zones are easy and comfortable to use, because the tractor driver does not need to create, actualize, and retain in his mind the treated zones map. He only needs to make a mental association between the map presented by the guidance system display and the land layout.

The system presented in this paper is another forward step in the simplification and comfort of the farmers’ work, because it allows them to always see the treated zones. They do not need to manage maps in their minds, nor do they need to make associations between the map and the terrain.

## 5. Conclusions

The use of AR technology in a tractor guidance system is viable and useful. It allows the tractor driver to see the treated zones in a natural way. The driver does not need to manage maps in his mind or make associations between a map and the terrain when no visible effects occur after a task, as occurs in many real agricultural scenarios.

The information contained in the treated zones maps of an assisted guidance system that uses AR technology must be complemented with information about the magnitude and sense of the steering wheel rotation required in order to follow the correct trajectory. In this way it is not necessary to have a very precise projection of the treated map over the field of vision. The presented system works fine over flat lands. A digital model of the terrain might be necessary in non flat lands.

The system could be used inside the tractor cabin for guidance tasks, but it can also be used outside, when the farmer is walking along the land. A future improvement of this AR system could provide it with the ability to introduce, show, and manage the spatial information of a *Geographic Information System* (GIS). The AR technology could then present the layers of the GIS over the farmer’s field of vision, and the use of the system would be very simple and natural.

## References

[1] Burdea G, Coiffet P (2003). Virtual Reality Technology.

[2] De Troyer O, Kleinermann F, Mansouri H, Pellens B, Bille W, Fomenko V (2007). Developing semantic VR-shops for e-Commerce. Virtual Reality.

[3] Seth A, Vance J, Oliver J (2010). Virtual reality for assembly methods prototyping: a review. Virtual reality.

[4] Moshell M (1993). Three views of virtual reality: virtual environments in the US military. Computer.

[5] Schultheis MT, Rizzo AA (2001). The Application of Virtual Reality Technology in Rehabilitation. Rehabil. Psychol.

[6] Yang JC, Chen CH, Chang Jeng M (2010). Integrating video-capture virtual reality technology into a physically interactive learning environment for English learning. Comput. Educ.

[7] Bimber O, Raskar R (2005). Spatial Augmented Reality: Merging Real and Virtual Worlds.

[8] Feiner S, Macintyre B, Seligmann D (1993). Knowledge-based augmented reality. Commun. ACM.

[9] Portalés C, Lerma JL, Navarro S (2010). Augmented reality and photogrammetry: A synergy to visualize physical and virtual city environments. ISPRS J. Photogramm. Remote Sens.

[10] Shin DH, Dunston PS (2008). Identification of application areas for Augmented Reality in industrial construction based on technology suitability. Autom. Constr.

[11] Ewers R, Schicho K, Undt G, Wanschitz F, Truppe M, Seemann R, Wagner A (2005). Basic research and 12 years of clinical experience in computer-assisted navigation technology: a review. Int. J. Oral Maxillofac. Surg.

[12] Henderson SJ, Feiner S Evaluating the benefits of augmented reality for task localization in maintenance of an armored personnel carrier turret.

[13] Jihyun O, Moon-Hyun L, Hanhoon P, Jong IIP, Jong-Sung K, Wookho S Efficient mobile museum guidance system using augmented reality.

[14] Kaufmann H, Schmalstieg D (2003). Mathematics and geometry education with collaborative augmented reality. Comput. Graph.-UK.

[15] Lin T-T, Hsiung Y-K, Hong G-L, Chang H-K, Lu F-M (2008). Development of a virtual reality GIS using stereo vision. Comput. Electron. Agric.

[16] Rovira-Más F, Zhang Q, Reid JF (2008). Stereo vision three-dimensional terrain maps for precision agriculture. Comput. Electron. Agric.

[17] Min S, Xiuwan C, Feizhou Z, Zheng H (2004). Augmented Reality Geographical Information System. Acta Scientiarum Naturalium Universitatis Pekinensis.

[18] Vidal NR, Vidal RA (2010). Augmented reality systems for weed economic thresholds applications. Planta Daninha.

[19] Board OAR, Shreiner D, Woo M, Neider J, Davis T (2007). OpenGL(R) Programming Guide: The Official Guide to Learning OpenGL(R), Version 21.

[20] Nagasaka Y, Umeda N, Kanetai Y, Taniwaki K, Sasaki Y (2004). Autonomous guidance for rice transplanting using global positioning and gyroscopes. Comput. Electron. Agric.

[21] Noguchi N, Ishii K, Terao H (1997). Development of an Agricultural Mobile Robot using a Geomagnetic Direction Sensor and Image Sensors. J. Agric. Eng. Res.

[22] Stoll A, Dieter Kutzbach H (2000). Guidance of a Forage Harvester with GPS. Precis. Agric.

[23] Samet H (1989). The Design and Analysis of Spatial Data Structures.

[24] Teejet Technologies Home Page http://www.teejet.com (accessed on 12 October 2010).

[25] Topcon Precision Agriculture Home Page http://www.topconpa.com (accessed on 12 October 2010).

